# Bis(1-methyl-4-oxoimidazolidin-2-iminium) diaqua­bis­(pyridine-2,4-dicarboxyl­ato-κ^2^ *N*,*O* ^2^)zincate(II) dihydrate

**DOI:** 10.1107/S1600536811008452

**Published:** 2011-03-12

**Authors:** Hossein Aghabozorg, Fatemeh Jafarbak, Masoud Mirzaei, Behrouz Notash

**Affiliations:** a Faculty of Chemistry, Islamic Azad University, North Tehran Branch, Tehran, Iran; b Department of Chemistry, School of Sciences, Ferdowsi University of Mashhad, Mashhad 917791436, Iran; c Department of Chemistry, Shahid Beheshti University, G. C., Evin, Tehran 1983963113, Iran

## Abstract

In the title compound, (C_4_H_8_N_3_O)_2_[Zn(C_7_H_3_NO_4_)_2_(H_2_O)_2_]·2H_2_O, the Zn^II^ ion is six-coordinated in a distorted octa­hedral geometry by two pyridine-2,4-dicarboxyl­ate (pydc) ligands in the equatorial plane and two water mol­ecules in the axial positions. The pydc ligands act as bidentate chelating ligands through one carboxyl­ate O atom and the pyridine N atom. Inter­molecular N—H⋯O, O—H⋯O and weak C—H⋯O hydrogen bonds stabilize the crystal structure.

## Related literature

For a review article on proton-transfer compounds, see: Aghabozorg *et al.* (2008*b* ▶). For related structures, see: Aghabozorg *et al.* (2008*a* ▶,*c* ▶); Attar Gharamaleki *et al.* (2009 ▶); Moghimi *et al.* (2004 ▶, 2005 ▶).
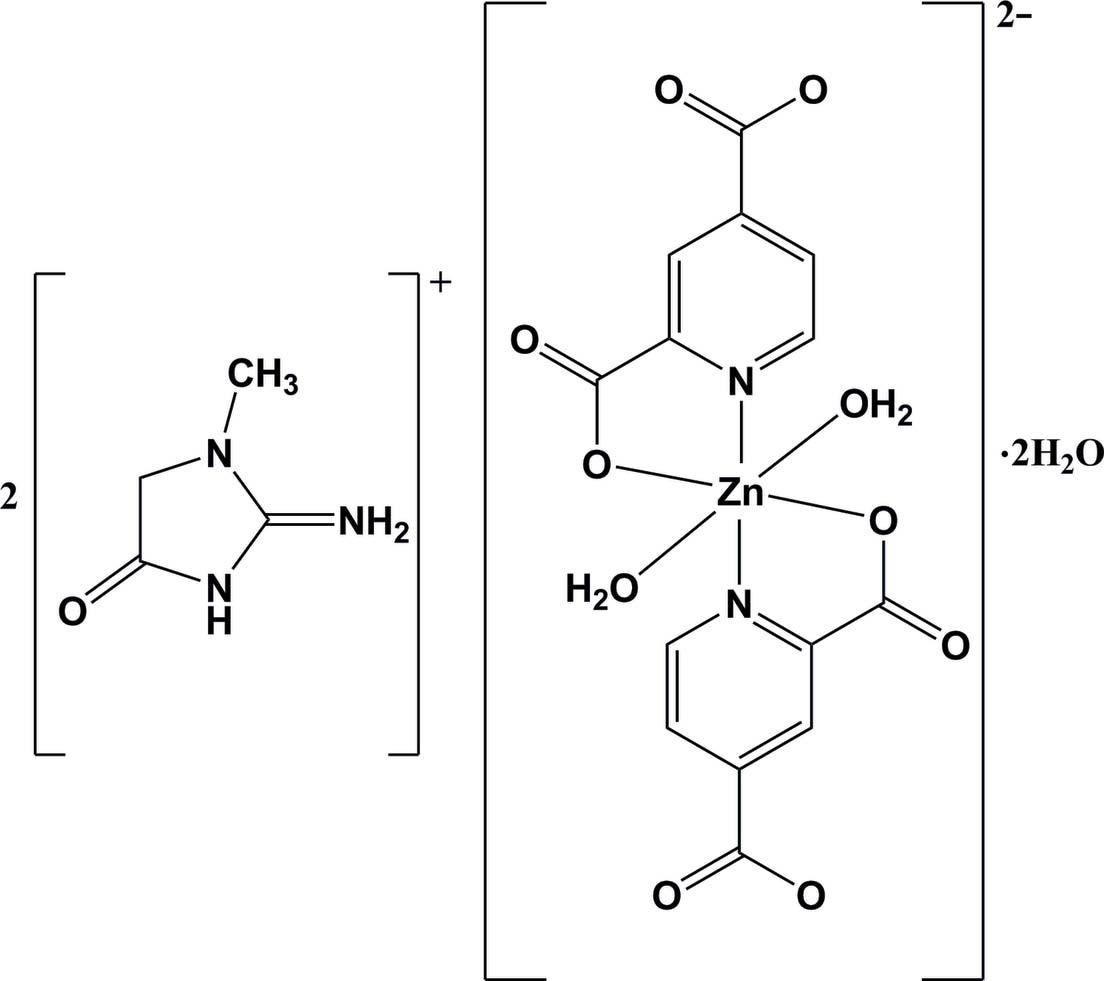


## Experimental

### 

#### Crystal data

(C_4_H_8_N_3_O)_2_[Zn(C_7_H_3_NO_4_)_2_(H_2_O)_2_]·2H_2_O
*M* *_r_* = 695.93
Triclinic, 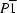

*a* = 5.3209 (11) Å
*b* = 8.3893 (17) Å
*c* = 16.621 (3) Å
α = 81.58 (3)°
β = 85.26 (3)°
γ = 74.09 (3)°
*V* = 705.1 (3) Å^3^
*Z* = 1
Mo *K*α radiation
μ = 0.96 mm^−1^
*T* = 298 K
0.30 × 0.20 × 0.15 mm

#### Data collection

Stoe IPDS-2 diffractometer
Absorption correction: numerical (*X-SHAPE* and *X-RED32*; Stoe & Cie, 2005 ▶) *T*_min_ = 0.793, *T*_max_ = 0.862
7743 measured reflections
3787 independent reflections
3206 reflections with *I* > 2σ(*I*)
*R*_int_ = 0.043

#### Refinement

*R*[*F* ^2^ > 2σ(*F* ^2^)] = 0.033
*wR*(*F* ^2^) = 0.090
*S* = 1.05
3787 reflections
226 parameters
1 restraint
H atoms treated by a mixture of independent and constrained refinement
Δρ_max_ = 0.46 e Å^−3^
Δρ_min_ = −0.48 e Å^−3^

### 

Data collection: *X-AREA* (Stoe & Cie, 2005 ▶); cell refinement: *X-AREA*; data reduction: *X-AREA*; program(s) used to solve structure: *SHELXS97* (Sheldrick, 2008 ▶); program(s) used to refine structure: *SHELXL97* (Sheldrick, 2008 ▶); molecular graphics: *ORTEP-3* (Farrugia, 1997 ▶); software used to prepare material for publication: *WinGX* (Farrugia, 1999 ▶).

## Supplementary Material

Crystal structure: contains datablocks I, global. DOI: 10.1107/S1600536811008452/hy2411sup1.cif

Structure factors: contains datablocks I. DOI: 10.1107/S1600536811008452/hy2411Isup2.hkl

Additional supplementary materials:  crystallographic information; 3D view; checkCIF report

## Figures and Tables

**Table 1:** Hydrogen-bond geometry (Å, °)

*D*—H⋯*A*	*D*—H	H⋯*A*	*D*⋯*A*	*D*—H⋯*A*
N3—H3⋯O3^i^	0.91 (3)	1.87 (3)	2.764 (2)	165 (3)
N4—H4*A*⋯O4^i^	0.86	1.86	2.706 (2)	168
N4—H4*B*⋯O2^ii^	0.86	1.92	2.771 (2)	169
O5—H5*A*⋯O4^i^	0.79 (2)	1.95 (3)	2.729 (2)	171 (3)
O5—H5*B*⋯O1^ii^	0.85 (3)	1.98 (3)	2.8088 (17)	165 (2)
O7—H7*A*⋯O3	0.73 (4)	2.32 (4)	2.968 (3)	148 (4)
O7—H7*B*⋯O6^iii^	0.82 (4)	2.26 (4)	2.993 (3)	149 (4)
C5—H5⋯O5^iv^	0.93	2.46	3.308 (2)	152
C11—H11*A*⋯O2^ii^	0.96	2.48	3.425 (3)	167

Symmetry codes: (i) 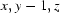; (ii) 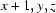; (iii) 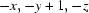; (iv) 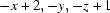.

**Fig. 1.**
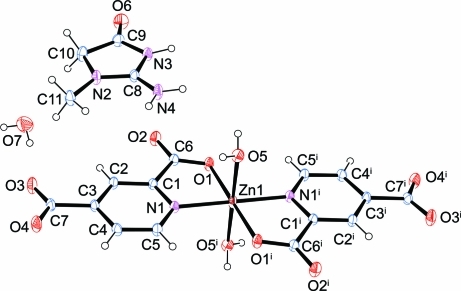
The molecular structure of the title compound, with displacement ellipsoids drawn at the 30% probability level. [Symmetry code: (i) -x+1, -y, -z+1.]

**Fig. 2.**
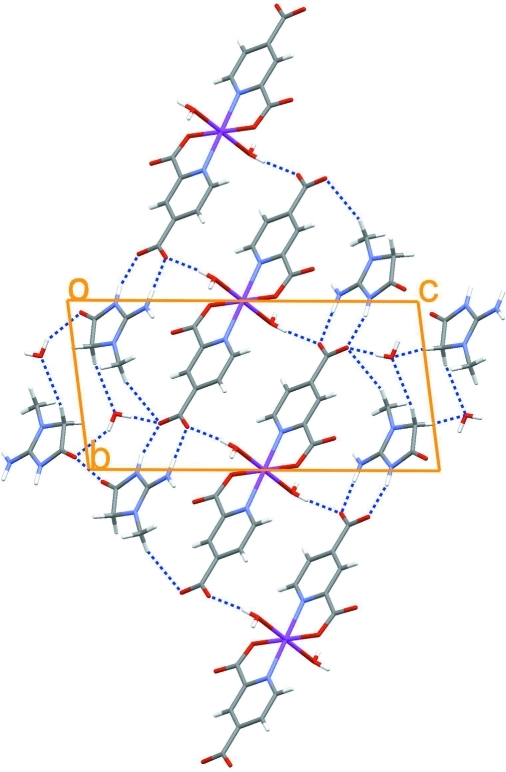
The packing diagram of the title compound. Intermolecular hydrogen bonds are shown as blue dashed lines.

## supplementary crystallographic information

### Comment

Many organic aromatic ligands and metal ions may aggregate into supramolecular networks using coordination and hydrogen bonds and π–π stacking interactions (Aghabozorg *et al.*, 2008*b*). Metal complexes of pyridine-(di)carboxylates possess versatile structural motifs, which finally aggregate to various supramolecular architectures with interesting properties. We have previously reported several compounds containing creatinine (creat), pyridine-2,6-dicarboxylic acid (pydcH_2_) and various metals, such as (creatH)(pydcH).H_2_O (Moghimi *et al.*, 2004), (creatH)_2_[Bi(pydc)_2_]_2_.4H_2_O (Moghimi *et al.*, 2005), (creatH)[Zn(pydc)(pydcH)].4H_2_O (Aghabozorg *et al.*, 2008*c*), (creatH)[Cr(pydc)_2_](pydcH_2_).6H_2_O (Aghabozorg *et al.*, 2008*a*) and (H_3_O)(creatH)[Ni(pydc)_2_].3H_2_O (Attar Gharamaleki *et al.*, 2009). For more details and related literature see our recent review article on proton-transfer compounds (Aghabozorg *et al.*, 2008*b*).

We describe here the crystal structure of the title compound. The compound contains a [Zn(pydc)_2_(H_2_O)_2_]^2-^ anion, two (creatH)^+^ cations and two uncoordinated water molecules (Fig. 1). In the anion, the Zn^II^ atom is six-coordinated by two N atoms and two O atoms from two pydc ligands and two water molecules, with the bond length range of 2.0819 (14)–2.1957 (15) Å. The coordination environment around Zn^II^ is distorted octahedral. Intermolecular N—H···O, O—H···O, and weak C—H···O hydrogen bonds play an important role in the stabilization of the crystal structure (Fig. 2 and Table 1).

### Experimental

The reaction of pyridine-2,4-dicarboxylic acid (83 mg, 0.5 mmol) in 10 ml distilled water, creatinine (56 mg, 0.5 mmol) in 5 ml distilled water and Zn(NO_3_)_2_.4H_2_O (65 mg, 0.25 mmol) in 10 ml distilled water in a 2:2:1 molar ratio gave colorless block crystals of the title compound after slow evaporation of the solvent at the room temperature. The crystals obtained were stable in air.

### Refinement

H atoms of water molecules and NH group were found in a difference Fourier map and refined isotropically. H5A was refined with a distance restraint of O—H = 0.79 (2) Å. H atoms on C atoms and NH_2_ group were positioned geometrically and refined as riding atoms, with C—H = 0.93 (CH), 0.97 (CH_2_) and 0.96 (CH_3_) Å and N—H = 0.86 Å and with *U*_iso_(H) = 1.2(1.5 for methyl)*U*_eq_(C, N).

### Crystal data

**Table:**

(C_4_H_8_N_3_O)_2_[Zn(C_7_H_3_NO_4_)_2_(H_2_O)_2_]·2H_2_O	*Z* = 1
*M**_r_* = 695.93	*F*(000) = 360.0
Triclinic, *P*1	*D*_x_ = 1.639 Mg m^−^^3^
Hall symbol: -P 1	Mo *K*α radiation, λ = 0.71073 Å
*a* = 5.3209 (11) Å	Cell parameters from 3787 reflections
*b* = 8.3893 (17) Å	θ = 2.5–29.2°
*c* = 16.621 (3) Å	µ = 0.96 mm^−^^1^
α = 81.58 (3)°	*T* = 298 K
β = 85.26 (3)°	Block, colorless
γ = 74.09 (3)°	0.30 × 0.20 × 0.15 mm
*V* = 705.1 (3) Å^3^	

### Data collection

**Table:**

Stoe IPDS-2 diffractometer	3787 independent reflections
Radiation source: fine-focus sealed tube	3206 reflections with *I* > 2σ(*I*)
graphite	*R*_int_ = 0.043
ω scans	θ_max_ = 29.2°, θ_min_ = 2.5°
Absorption correction: numerical (*X-SHAPE* and *X-RED32*; Stoe & Cie, 2005)	*h* = −7→7
*T*_min_ = 0.793, *T*_max_ = 0.862	*k* = −11→11
7743 measured reflections	*l* = −18→22

### Refinement

**Table:**

Refinement on *F*^2^	Primary atom site location: structure-invariant direct methods
Least-squares matrix: full	Secondary atom site location: difference Fourier map
*R*[*F*^2^ > 2σ(*F*^2^)] = 0.033	Hydrogen site location: inferred from neighbouring sites
*wR*(*F*^2^) = 0.090	H atoms treated by a mixture of independent and constrained refinement
*S* = 1.05	*w* = 1/[σ^2^(*F*_o_^2^) + (0.059*P*)^2^] where *P* = (*F*_o_^2^ + 2*F*_c_^2^)/3
3787 reflections	(Δ/σ)_max_ = 0.001
226 parameters	Δρ_max_ = 0.46 e Å^−^^3^
1 restraint	Δρ_min_ = −0.48 e Å^−^^3^

### Fractional atomic coordinates and isotropic or equivalent isotropic displacement parameters (Å^2^)

**Table:**

	*x*	*y*	*z*	*U*_iso_*/*U*_eq_	
Zn1	0.5000	0.0000	0.5000	0.02707 (9)	
O1	0.2451 (2)	0.02688 (14)	0.40534 (7)	0.0301 (2)	
O2	0.1143 (3)	0.18980 (16)	0.28950 (8)	0.0395 (3)	
O3	0.4093 (3)	0.72611 (16)	0.22032 (9)	0.0432 (3)	
O4	0.7237 (3)	0.74523 (17)	0.29581 (9)	0.0462 (4)	
O5	0.8365 (2)	−0.13001 (16)	0.42711 (8)	0.0325 (3)	
O6	0.1878 (3)	0.07267 (19)	0.03511 (9)	0.0476 (3)	
O7	0.0855 (4)	0.6692 (3)	0.09580 (15)	0.0641 (5)	
N1	0.5314 (2)	0.22674 (15)	0.43582 (8)	0.0239 (2)	
N2	0.6139 (3)	0.25207 (17)	0.11654 (9)	0.0313 (3)	
N3	0.4814 (3)	0.02055 (17)	0.13579 (9)	0.0305 (3)	
N4	0.7948 (3)	0.03970 (18)	0.22119 (10)	0.0379 (4)	
H4B	0.8947	0.0943	0.2359	0.045*	
H4A	0.7965	−0.0579	0.2462	0.045*	
C1	0.3894 (3)	0.27280 (17)	0.36902 (9)	0.0228 (3)	
C2	0.3856 (3)	0.41957 (18)	0.31741 (9)	0.0266 (3)	
H2	0.2819	0.4505	0.2724	0.032*	
C3	0.5398 (3)	0.51957 (17)	0.33430 (9)	0.0252 (3)	
C4	0.6886 (3)	0.46909 (18)	0.40272 (10)	0.0282 (3)	
H4	0.7951	0.5326	0.4151	0.034*	
C5	0.6774 (3)	0.32360 (19)	0.45239 (10)	0.0277 (3)	
H5	0.7746	0.2921	0.4988	0.033*	
C6	0.2358 (3)	0.15364 (18)	0.35239 (9)	0.0251 (3)	
C7	0.5555 (3)	0.67753 (19)	0.27867 (10)	0.0302 (3)	
C8	0.6413 (3)	0.10458 (19)	0.16135 (10)	0.0275 (3)	
C9	0.3457 (3)	0.1124 (2)	0.07056 (11)	0.0322 (3)	
C10	0.4348 (3)	0.2712 (2)	0.05257 (11)	0.0348 (4)	
H10A	0.5227	0.2790	−0.0008	0.042*	
H10B	0.2887	0.3694	0.0555	0.042*	
C11	0.7495 (4)	0.3769 (2)	0.12415 (12)	0.0360 (4)	
H11A	0.8693	0.3364	0.1673	0.054*	
H11B	0.6247	0.4781	0.1362	0.054*	
H11C	0.8445	0.3988	0.0739	0.054*	
H3	0.453 (6)	−0.081 (4)	0.155 (2)	0.076 (9)*	
H7A	0.113 (8)	0.685 (5)	0.136 (2)	0.085 (13)*	
H5B	0.952 (5)	−0.078 (3)	0.4112 (15)	0.048 (7)*	
H7B	0.025 (7)	0.763 (5)	0.072 (2)	0.083 (11)*	
H5A	0.818 (6)	−0.174 (3)	0.3900 (14)	0.060 (8)*	

### Atomic displacement parameters (Å^2^)

**Table:**

	*U*^11^	*U*^22^	*U*^33^	*U*^12^	*U*^13^	*U*^23^
Zn1	0.03224 (15)	0.02409 (13)	0.02854 (15)	−0.01705 (9)	−0.01220 (10)	0.00935 (9)
O1	0.0344 (6)	0.0286 (5)	0.0326 (6)	−0.0204 (4)	−0.0115 (5)	0.0074 (4)
O2	0.0513 (7)	0.0399 (6)	0.0359 (7)	−0.0273 (6)	−0.0237 (6)	0.0088 (5)
O3	0.0569 (8)	0.0353 (6)	0.0435 (7)	−0.0273 (6)	−0.0233 (6)	0.0156 (5)
O4	0.0700 (9)	0.0397 (7)	0.0419 (7)	−0.0400 (7)	−0.0218 (7)	0.0124 (5)
O5	0.0335 (6)	0.0362 (6)	0.0326 (6)	−0.0182 (5)	−0.0065 (5)	−0.0004 (5)
O6	0.0467 (8)	0.0542 (8)	0.0483 (8)	−0.0211 (6)	−0.0229 (6)	−0.0009 (7)
O7	0.0781 (13)	0.0550 (10)	0.0624 (12)	−0.0174 (9)	−0.0188 (10)	−0.0093 (9)
N1	0.0256 (6)	0.0233 (5)	0.0251 (6)	−0.0117 (4)	−0.0077 (5)	0.0029 (4)
N2	0.0348 (7)	0.0267 (6)	0.0343 (7)	−0.0152 (5)	−0.0096 (6)	0.0081 (5)
N3	0.0394 (7)	0.0280 (6)	0.0295 (7)	−0.0187 (5)	−0.0106 (6)	0.0031 (5)
N4	0.0529 (9)	0.0315 (7)	0.0369 (8)	−0.0264 (6)	−0.0225 (7)	0.0119 (6)
C1	0.0247 (6)	0.0227 (6)	0.0242 (7)	−0.0125 (5)	−0.0057 (5)	0.0013 (5)
C2	0.0323 (7)	0.0246 (6)	0.0254 (7)	−0.0140 (6)	−0.0099 (6)	0.0053 (5)
C3	0.0301 (7)	0.0208 (6)	0.0268 (7)	−0.0121 (5)	−0.0059 (6)	0.0028 (5)
C4	0.0328 (8)	0.0247 (7)	0.0321 (8)	−0.0164 (6)	−0.0092 (6)	0.0013 (6)
C5	0.0313 (7)	0.0266 (7)	0.0286 (7)	−0.0141 (6)	−0.0130 (6)	0.0041 (6)
C6	0.0269 (7)	0.0254 (6)	0.0273 (7)	−0.0149 (5)	−0.0071 (6)	0.0010 (5)
C7	0.0424 (9)	0.0228 (6)	0.0296 (8)	−0.0173 (6)	−0.0054 (6)	0.0024 (6)
C8	0.0335 (8)	0.0254 (7)	0.0269 (7)	−0.0152 (6)	−0.0047 (6)	0.0024 (6)
C9	0.0321 (8)	0.0349 (8)	0.0308 (8)	−0.0108 (6)	−0.0080 (6)	−0.0005 (6)
C10	0.0338 (8)	0.0338 (8)	0.0354 (9)	−0.0111 (6)	−0.0113 (7)	0.0094 (7)
C11	0.0402 (9)	0.0273 (7)	0.0435 (10)	−0.0180 (6)	−0.0069 (7)	0.0055 (7)

### Geometric parameters (Å, °)

**Table:**

Zn1—N1	2.0819 (14)	N3—H3	0.91 (4)
Zn1—O1	2.1087 (13)	N4—C8	1.298 (2)
Zn1—O5	2.1957 (15)	N4—H4B	0.8600
O1—C6	1.2696 (18)	N4—H4A	0.8600
O2—C6	1.2303 (19)	C1—C2	1.390 (2)
O3—C7	1.244 (2)	C1—C6	1.520 (2)
O4—C7	1.254 (2)	C2—C3	1.393 (2)
O5—H5B	0.85 (3)	C2—H2	0.9300
O5—H5A	0.79 (2)	C3—C4	1.387 (2)
O6—C9	1.207 (2)	C3—C7	1.519 (2)
O7—H7A	0.74 (4)	C4—C5	1.383 (2)
O7—H7B	0.82 (4)	C4—H4	0.9300
N1—C5	1.3367 (19)	C5—H5	0.9300
N1—C1	1.3437 (19)	C9—C10	1.514 (3)
N2—C8	1.325 (2)	C10—H10A	0.9700
N2—C10	1.449 (2)	C10—H10B	0.9700
N2—C11	1.449 (2)	C11—H11A	0.9600
N3—C9	1.368 (2)	C11—H11B	0.9600
N3—C8	1.370 (2)	C11—H11C	0.9600
			
N1—Zn1—N1^i^	180.000 (1)	C1—C2—H2	120.6
N1—Zn1—O1	79.39 (5)	C3—C2—H2	120.6
N1^i^—Zn1—O1	100.61 (5)	C4—C3—C2	118.38 (13)
N1—Zn1—O1^i^	100.61 (5)	C4—C3—C7	119.66 (14)
N1^i^—Zn1—O1^i^	79.39 (5)	C2—C3—C7	121.92 (14)
O1—Zn1—O1^i^	180.00 (4)	C5—C4—C3	119.50 (14)
N1—Zn1—O5	89.26 (6)	C5—C4—H4	120.2
N1^i^—Zn1—O5	90.74 (6)	C3—C4—H4	120.2
O1—Zn1—O5	91.70 (5)	N1—C5—C4	122.15 (14)
O1^i^—Zn1—O5	88.30 (5)	N1—C5—H5	118.9
N1—Zn1—O5^i^	90.74 (6)	C4—C5—H5	118.9
N1^i^—Zn1—O5^i^	89.26 (6)	O2—C6—O1	126.60 (14)
O1—Zn1—O5^i^	88.30 (5)	O2—C6—C1	117.01 (13)
O1^i^—Zn1—O5^i^	91.70 (5)	O1—C6—C1	116.38 (13)
O5—Zn1—O5^i^	180.000 (1)	O3—C7—O4	125.95 (15)
C6—O1—Zn1	114.83 (9)	O3—C7—C3	118.71 (14)
Zn1—O5—H5B	116.8 (17)	O4—C7—C3	115.32 (14)
Zn1—O5—H5A	121 (2)	N4—C8—N2	127.93 (15)
H5B—O5—H5A	106 (3)	N4—C8—N3	121.60 (14)
H7A—O7—H7B	103 (4)	N2—C8—N3	110.47 (13)
C5—N1—C1	118.96 (13)	O6—C9—N3	126.13 (17)
C5—N1—Zn1	127.89 (10)	O6—C9—C10	127.74 (16)
C1—N1—Zn1	113.12 (10)	N3—C9—C10	106.12 (14)
C8—N2—C10	110.12 (14)	N2—C10—C9	102.65 (13)
C8—N2—C11	127.22 (14)	N2—C10—H10A	111.2
C10—N2—C11	122.56 (14)	C9—C10—H10A	111.2
C9—N3—C8	110.51 (14)	N2—C10—H10B	111.2
C9—N3—H3	118 (2)	C9—C10—H10B	111.2
C8—N3—H3	131 (2)	H10A—C10—H10B	109.2
C8—N4—H4B	120.0	N2—C11—H11A	109.5
C8—N4—H4A	120.0	N2—C11—H11B	109.5
H4B—N4—H4A	120.0	H11A—C11—H11B	109.5
N1—C1—C2	122.12 (13)	N2—C11—H11C	109.5
N1—C1—C6	116.08 (12)	H11A—C11—H11C	109.5
C2—C1—C6	121.80 (13)	H11B—C11—H11C	109.5
C1—C2—C3	118.85 (13)		
			
N1—Zn1—O1—C6	3.82 (11)	C3—C4—C5—N1	1.5 (3)
N1^i^—Zn1—O1—C6	−176.18 (11)	Zn1—O1—C6—O2	175.35 (14)
O5—Zn1—O1—C6	−85.11 (12)	Zn1—O1—C6—C1	−5.02 (18)
O5^i^—Zn1—O1—C6	94.89 (12)	N1—C1—C6—O2	−176.70 (15)
O1—Zn1—N1—C5	−179.66 (15)	C2—C1—C6—O2	2.4 (2)
O1^i^—Zn1—N1—C5	0.34 (15)	N1—C1—C6—O1	3.6 (2)
O5—Zn1—N1—C5	−87.80 (14)	C2—C1—C6—O1	−177.22 (14)
O5^i^—Zn1—N1—C5	92.20 (14)	C4—C3—C7—O3	−175.83 (16)
O1—Zn1—N1—C1	−1.69 (11)	C2—C3—C7—O3	6.3 (3)
O1^i^—Zn1—N1—C1	178.31 (11)	C4—C3—C7—O4	5.8 (2)
O5—Zn1—N1—C1	90.17 (11)	C2—C3—C7—O4	−172.14 (17)
O5^i^—Zn1—N1—C1	−89.83 (11)	C10—N2—C8—N4	176.19 (18)
C5—N1—C1—C2	−1.2 (2)	C11—N2—C8—N4	−0.2 (3)
Zn1—N1—C1—C2	−179.41 (12)	C10—N2—C8—N3	−3.2 (2)
C5—N1—C1—C6	177.91 (14)	C11—N2—C8—N3	−179.64 (17)
Zn1—N1—C1—C6	−0.26 (17)	C9—N3—C8—N4	−178.09 (16)
N1—C1—C2—C3	1.9 (2)	C9—N3—C8—N2	1.4 (2)
C6—C1—C2—C3	−177.24 (14)	C8—N3—C9—O6	−179.20 (18)
C1—C2—C3—C4	−0.8 (2)	C8—N3—C9—C10	1.0 (2)
C1—C2—C3—C7	177.16 (15)	C8—N2—C10—C9	3.57 (19)
C2—C3—C4—C5	−0.8 (2)	C11—N2—C10—C9	−179.80 (16)
C7—C3—C4—C5	−178.81 (15)	O6—C9—C10—N2	177.51 (19)
C1—N1—C5—C4	−0.5 (2)	N3—C9—C10—N2	−2.67 (19)
Zn1—N1—C5—C4	177.40 (12)		

Symmetry codes: (i) −*x*+1, −*y*, −*z*+1.

### Hydrogen-bond geometry (Å, °)

**Table:**

*D*—H···*A*	*D*—H	H···*A*	*D*···*A*	*D*—H···*A*
N3—H3···O3^ii^	0.91 (3)	1.87 (3)	2.764 (2)	165 (3)
N4—H4A···O4^ii^	0.86	1.86	2.706 (2)	168
N4—H4B···O2^iii^	0.86	1.92	2.771 (2)	169
O5—H5A···O4^ii^	0.79 (2)	1.95 (3)	2.729 (2)	171 (3)
O5—H5B···O1^iii^	0.85 (3)	1.98 (3)	2.8088 (17)	165 (2)
O7—H7A···O3	0.73 (4)	2.32 (4)	2.968 (3)	148 (4)
O7—H7B···O6^iv^	0.82 (4)	2.26 (4)	2.993 (3)	149 (4)
C5—H5···O5^v^	0.93	2.46	3.308 (2)	152
C11—H11A···O2^iii^	0.96	2.48	3.425 (3)	167

Symmetry codes: (ii) *x*, *y*−1, *z*; (iii) *x*+1, *y*, *z*; (iv) −*x*, −*y*+1, −*z*; (v) −*x*+2, −*y*, −*z*+1.
